# Purification and characterization of a cold-active myrosinase from marine *Pseudomonas oleovorans* SuMy07: Cold-active myrosinase from SuMy07

**DOI:** 10.3724/abbs.2023051

**Published:** 2023-03-28

**Authors:** Zhifa Huang, Nannan Liu, Yaowei Fang, Xiaoyue Hou, Guang Yang, Jing Lu, Haoyu Mi, Qinwen Ye, Rongjun Zhu, Shu Liu

**Affiliations:** 1 Jiangsu Key Laboratory of Marine Bioresources and Environment Jiangsu Ocean University Lianyungang 222005 China; 2 Co-Innovation Center of Jiangsu Marine Bio-industry Technology Jiangsu Ocean University Lianyungang 222005 China; 3 School of Food Science and Engineering Jiangsu Ocean University Lianyungang 222005 China; 4 Jiangsu Marine Resources Development Research Institute Lianyungang 222005 China

Myrosinase (EC 3.2.1.147), also known as β-thioglucoside glucohydrolase, was first discovered in mustard seeds. Myrosinase hydrolyzes glucosinolate to produce D-glucose and an unstable aglycon, which upon rearrangement yields metabolites such as isothiocyanate, sulfate, acetonitrile, and thiocyanate. Isothiocyanate has a wide range of biological functions [1]. Previous studies have shown that isothiocyanate has insect-repellant and antibacterial effects [2]. Isothiocyanate also has anti-inflammatory and antioxidant properties, making it a potential anticancer compound [ 3, 4] . Sulforaphane is one of the most representative isothiocyanate substances against numerous cancers. Myrosinase extraction is technically challenging, although myrosinase metabolites have a variety of uses. At present, myrosinase is primarily extracted from white mustard seeds after a time-consuming and expensive preparation. Therefore, the search for myrosinase that can hydrolyze glucosinolates to produce isothiocyanate efficiently and economically has become an urgent task. Microbes can secrete myrosinase with rapid growth, lack of seasonal variation, simple intensive cultivation, and simple product extraction. Considering that seawater includes a large number of glucosinolates, the complex ecosystem of the marine environment can serve as a breeding ground for microorganisms that produce myrosinase. In the present study, the myrosinase-producing strain SuMy07 in the marine environment was screened out, and its enzyme composition and potential application were investigated. As a novel marine strain capable of producing myrosinase, SuMy07 could be an excellent candidate for the preparation of isothiocyanate.

Bacteria will face some physical and chemical variations in the marine environment, and a complex, constantly changing environment can provide the right conditions for microorganisms to produce distinct secondary metabolites. Therefore, bacteria that produce myrosinase can be found in large numbers in the ocean. Mud samples were collected at 15 different locations in Haizhou Bay (Lianyungang, China). Sinigrin hydrate (chromatography grade, >98.0%; Sigma-Aldrich, St Louis, USA) was used as a substrate for the reaction. After initial screening and rescreening, 12 strains were selected due to their obvious precipitation circle on the screening medium. These strains were chosen for enzyme activity determination ( Supplementary Table S1). To identify the strains that hydrolyze the sinigrin hydrate the most, high **-**performance liquid chromatography was used to analyze the sinigrin hydrate consumption ( Figure 1A). SuMy07 was chosen for the following study because it had the highest consumption of sinigrin (0.52 mg/mL). Physiological and biochemical identification of SuMy07 was carried out. SuMy07 formed round, milky white, flat, and opaque colonies on an agar plate. Gram staining identified the strain as a Gram-negative bacterium. A comparison of the gene sequence with the NCBI database revealed 99.85% homology between SuMy07 and *Pseudomonas oleovorans*. The neighbour-joining method in MEGA 7.0 was used to construct a phylogenetic tree containing the sequenced strain and 14 sequences with high similarity to the sequenced strain with a total of 1000 bootstrap iterations ( Figure 1B). Combined with NCBI database comparison and phylogenetic tree construction results, the strain was identified as *P*. *oleovorans*. To our knowledge, our study of myrosinase-producing *Pseudomonas* had sufficient novelty. Plant-derived and insect-derived myrosinases were dominant in previous studies, and only a few studies were conducted on microbe-derived myrosinases. *Citrobacter* sp. WYE1, which can produce myrosinase, was first isolated from the soil in the UK [5]. *Shewanella baltica*, which can produce novel myrosinase, was described in 2022 [6]. Our discovery of the myrosinase-producing marine bacterium SuMy07 is a solid complement to the microbial sources of myrosinase.

**Figure 1:**
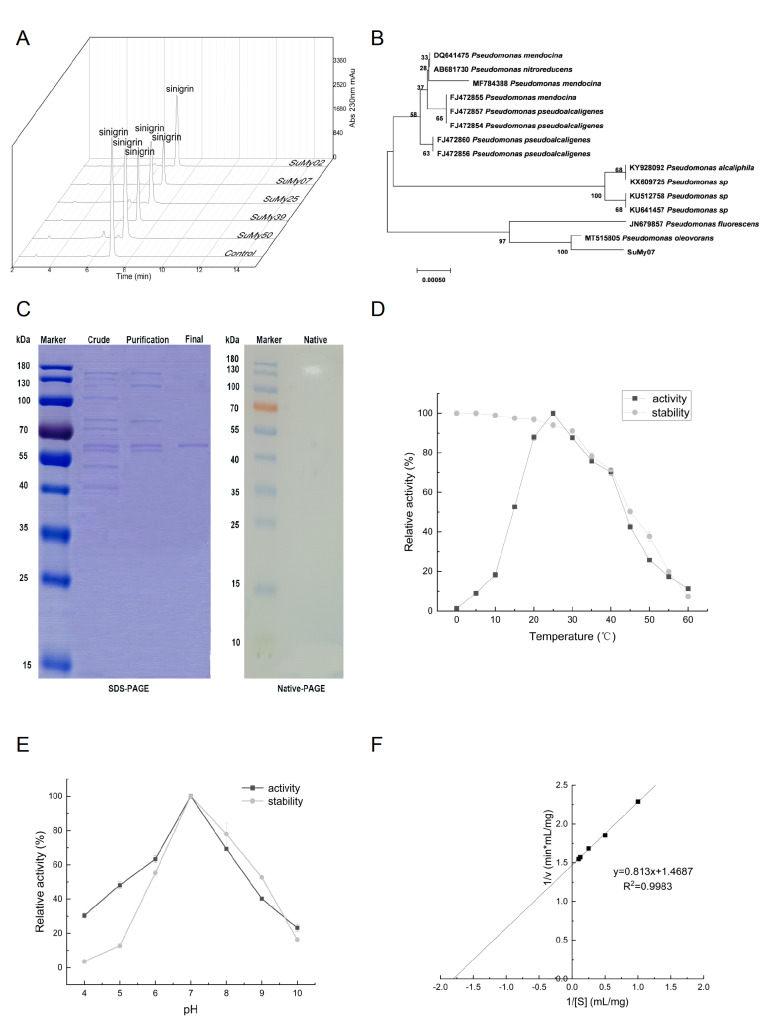
Figure 1 Identification of SuMy07 and characterization of the enzymatic properties of myrosinase (A) High-performance liquid chromatography analysis at 230 nm of the sinigrin content in the fermentation broth. (B) Phylogenetic tree constructed based on strain SuMy07 16S rDNA sequence. (C) SDS-PAGE analysis for the purification of myrosinase and the native-PAGE analysis of myrosinase. Crude: crude enzyme; purification: ultrafilter tube interception; final: gel filtration; and native: native-PAGE analysis. (D) The effect of temperature on enzyme activity and stability. (E) The effect of pH on enzyme activity and stability. (F) Determination of K m and V max values using double-reciprocal plot.

The purified protein was obtained by ultrafiltration, ammonium sulfate precipitation, and gel filtration chromatography ( Supplementary Table S2). Ultrafiltration tubes (100 kDa, 50 kDa, 30 kDa, and 10 kDa) were used for initial purification, and the enzymatic activity in ultrafiltration tubes with a molecular weight of 100 kDa was the highest, reaching 45.57 U/mg. The crude enzyme solution with the highest enzymatic activity was precipitated with different concentrations of ammonium sulfate solution (0%–20%, 20%–40%, 60%–80%, and 80%–100%) to purify the crude enzyme solution. The enzymatic activity was the highest when the concentration of ammonium sulfate was 0%–20%, which was 59.73 U/mg. The protein with the highest enzymatic activity was purified by gel filtration using a Superdex 200 Increase 10/300 column (GE Healthcare, Wisconsin, USA), and the enzymatic activity of the isolated protein reached 267.00 U/mg. The molecular weight of the purified protein was determined by SDS-PAGE and native PAGE. The results are shown in Figure 1C. The myrosinase of SuMy07 is composed of two subunits with a molecular weight of 65 kDa. Previous reports showed that the myrosinases found in plants and aphids belong to the GH1 family, while bacteria such as *Rahnella inusitata* belong to the GH3 family [7]. Therefore, the gene sequence encoding myrosinase in bacteria differs from that in plants or insects. This variation can lead to difference in the properties of the encoded proteins. According to a study of myrosinase from horseradish, the molecular weight of the myrosinase monomer is 65 kDa, and that of the natural protein is 130 kDa. The subunits of broccoli myrosinase have a molecular mass of 50–55 kDa. The myrosinase from *Citrobacter* *sp*. WYE1 has a molecular weight of approximately 66 kDa [5]. According to previous studies, myrosinase generally consists of two identical polypeptides of 55–65 kDa, which are highly glycosylated, resulting in a dimeric protein with a native molecular weight of 120–150 kDa [8].

After the purified myrosinase was obtained, its enzymatic properties were investigated. The influence of temperature on myrosinase activity is shown in Figure 1D. Myrosinase had an optimum activity temperature of 25°C, which remained relatively high between 20°C and 40°C. The activity declined rapidly at temperatures higher than 40°C. The effect of pH on myrosinase activity is shown in Figure 1E. The myrosinase activity steadily increased from pH 5.0 to the maximum pH 7.0, and the tolerance of myrosinase to different pH values showed that its enzymatic activity was extremely low after reaching pH 9.0. Consequently, myrosinase can efficiently maintain its activity in a weakly acidic environment to a neutral environment. The optimum temperature for plant-derived myrosinase is between 30°C and 60°C, for example, the optimum temperatures for myrosinases from white mustard seed and horseradish are 55°C and 45°C, respectively, which is higher than that for microbial-derived myrosinase. In the present study, the optimal reaction temperature of SuMy07 myrosinase was low, indicating its wide range of applications in industrial production. In industrial manufacturing, cold-active enzymes are necessary because they can save a significant amount of energy. For example, using cold-adapted enzymes in food processing can avoid high-temperature processing steps, help retain flavour and improve nutritional value [9]. Strain SuMy07 was derived from the ocean, and the low-temperature and weakly alkaline marine environment primarily caused the optimum temperature of myrosinase to be 25°C and the optimum pH to be 7.0. The effect of metal ions on myrosinase activity was shown in Supplementary Table S3. The myrosinase activity was primarily inhibited by Al ^3+^ and Pb ^2+^, reaching more than 50%. Additionally, it was suppressed by Fe ^3+^, Ba ^2+^, Cu ^2+^, Mg ^2+^, and K ^+^ to a certain extent but primarily promoted by Ca ^2+^ to up to 113.11%. Analysis of the effects of metal ions on myrosinase activity showed that most metal ions had inhibitory effects on myrosinase activity. Metal ions that hinder the activity of myrosinase must be avoided in practical applications. At pH 7.0 and 25°C, the purified myrosinase showed a *V*_max_ of 1.7 mM/min and a *K*_m_ of 1.4 mM using sinigrin hydrate as the substrate ( Figure 1F). Studies have shown that myrosinases from white mustard seed and horseradish have *K*_m_ values of 0.15 mM and 0.128 mM, respectively [8]. The myrosinase of SuMy07 exhibited a lower substrate affinity than that generated from plants, indicating that SuMy07 is a new myrosinase with different enzymatic properties from previously identified myrosinases, such as molecular weight of the enzyme, optimal temperature and pH, and affinity of the enzyme to the substrate.

Under appropriate conditions, glucoraphanin will be hydrolyzed by myrosinase to produce sulforaphane ( Figure 2A). The newly identified myrosinase from SuMy07 was investigated for its ability to hydrolyze glucoraphanin to produce sulforaphane by high-performance liquid chromatography (HPLC). The system was injected with 0.1–1.0 mg/mL sulforaphane standard (Sigma-Aldrich, St Louis, USA HPLC, >98.0%) to create a standard curve between the content and peak region. The mobile phases included acetonitrile and water. Glucoraphanin (chromatography grade, >98.0%; Sigma-Aldrich) and enzyme solution were mixed and incubated for an hour and filtered, and the peak time was determined by HPLC. The results are shown in Figure 2B. The peaks for the standard and sample occurred at approximately 9 min. No peak was observed in the control group, indicating that the glucoraphanin in the solution was hydrolyzed to sulforaphane in the presence of SuMy07. A total of 0.483 mg of sulforaphane was generated from the co-reaction with the crude extract of glucoraphanin for 1.25 h. This finding verified the existence of this myrosinase strain and its potential to hydrolyze glucoraphanin into sulforaphane. Examination of the effect of reaction time on sulforaphane yield revealed that the amount of sulforaphane generated reached a maximum of 0.483 g/L at 1.25 h of reaction. Later, a modest drop in sulforaphane production occurred, possibly due to the disintegration of sulforaphane caused by the attack of hydroxyl groups in water ( Figure 2C). Myrosinase hydrolyzed 14.07 mg (32.2 μmol) of glucoraphanin in 10 mL of crude radish seed extract to produce 4.83 mg (27.2 μmol) of sulforaphane in approximately 1.25 h at the ideal temperature. The sulforaphane output and conversion rates were 21.76 μmol/h and 84.47%, respectively. This result differed from the results obtained by Wang *et al*. [10], who reported 23.2 μmol/h and 98.69% total yield and conversion rate of the allomyrosinase gene introduced into yeast, respectively. However, they studied a myrosinase gene from *Arabidopsis thaliana* that was heterogeneously transferred into yeast. Therefore, as an original strain, the myrosinase of SuMy07 in the present work has great application potential for sulforaphane conversion.

**Figure 2:**
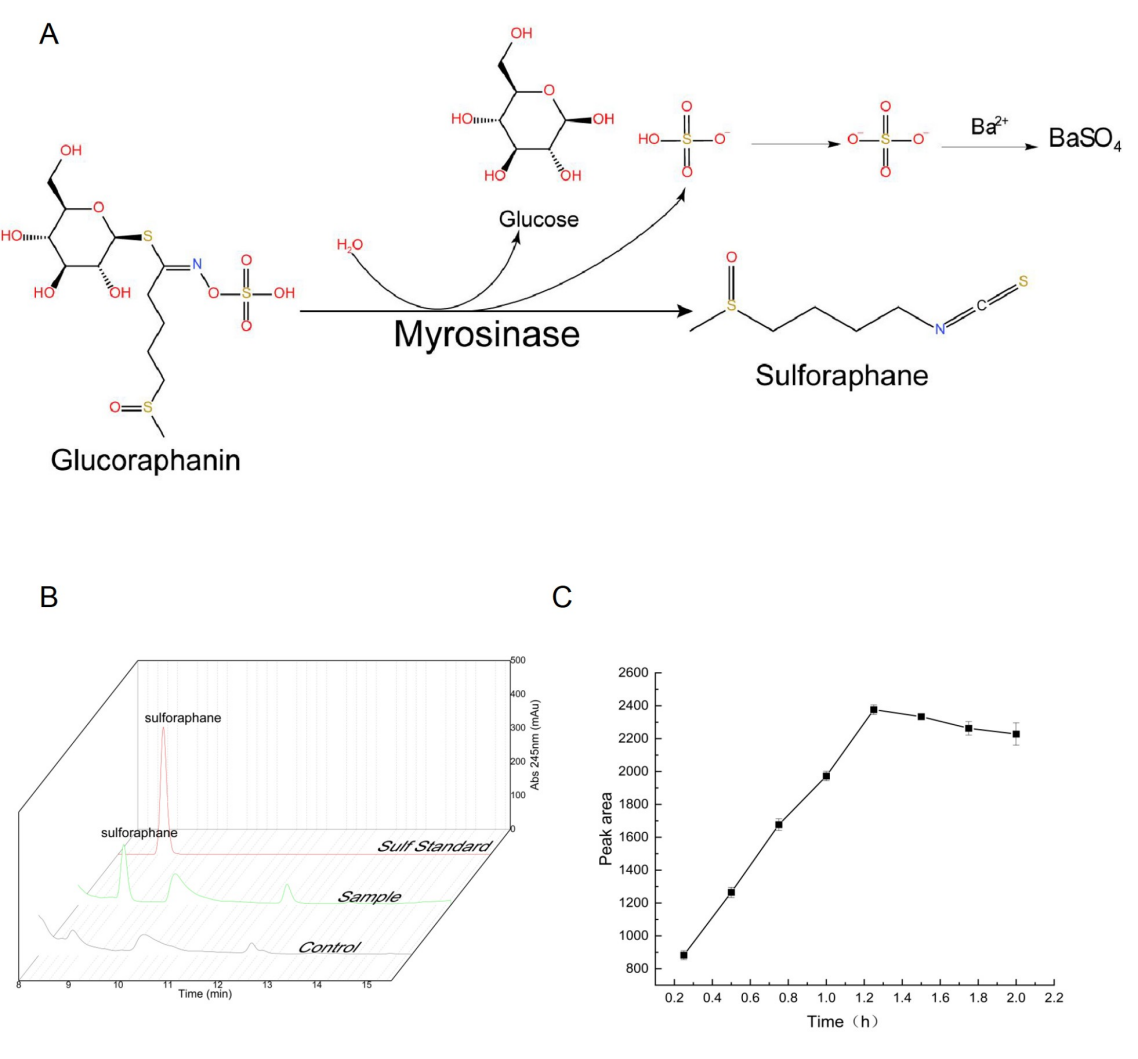
Figure 2 The myrosinase of SuMy07 hydrolyzes glucoraphanin to produce sulforaphane (A) Hydrolysis of glucoraphanin by myrosinase. (B) Comparison of the production of sulforaphane. Sul standard: sulforaphane standard; sample: myrosinase+glucoraphanin; and control: glucoraphanin. (C) Peak area (sulforaphane concentration) at different reaction time points.

In summary, we isolated a marine bacterium *Pseudomonas oleovorans* SuMy07 from the ocean, which is capable of producing a novel cold-active myrosinase. This myrosinase can hydrolyze glucoraphanin to sulforaphane. This work advances the research of marine microbes, establishes and clarifies the analysis of marine myrosinase-producing strains, and provides novel insights into microbial myrosinase production.

## Supporting information

22667Supplementary_Table_

## Supplementary Data

Supplementary data is available at *Acta Biochimica et Biophysica Sinica* online.
