# Supplementary material for: Purification and characterization of a cold-active myrosinase from marine Pseudomonas oleovorans SuMy07 : Cold-active myrosinase from SuMy07
Source: Acta Biochim Biophys Sin (Shanghai). 2023 Mar 28;55(4):695–9. doi: 10.3724/abbs.2023051 (PMC10195144; doi:10.3724/abbs.2023051)
Supplement: 22667Supplementary_Table_ [file 22667Supplementary_Table_.pdf]

Supplementary Table S1. Enzyme activity of different strains

Strain Number	Enzymatic activity (U)
SuMy02	48.67 ± 1.20
SuMy04	42.00 ± 0.36
SuMy07	93.57 ± 0.78
SuMy08	32.77 ± 0.12
SuMy14	32.70 ± 0.26
SuMy25	34.23 ± 1.03
SuMy36	41.07 ± 0.86
SuMy37	32.53 ± 1.25
SuMy39	33.03 ± 0.31
SuMy45	48.70 ± 0.69
SuMy49	32.70 ± 1.20
SuMy50	27.63 ± 0.75

Supplementary Table S2. Purification of myrosinase

Step of purification	Total activity (U/mL)	Total protein (mg/mL)	Specific activity (U/mg)	Yield (%)
Crude enzyme	52.67	1.52	34.65	100.00
Ultrafiltration tube for ultrafiltration	36.91	0.81	45.57	70.08
Ammonium sulfate precipitation	35.24	0.59	59.73	66.91
Gel filtration chromatography	29.37	0.11	267.00	55.76

Supplementary Table S3. The effect of metal ions on myrosinase activity

Metal ions (10 mM)	Enzymatic activity (U)	Relative activity (%)
Contrast	56.37 ± 1.02	100.00
Mg^{2+}	45.38 ± 0.63	80.50
Zn^{2+}	56.81 ± 0.36	100.78
Cu^{2+}	30.27 ± 0.98	53.70
Ba^{2+}	29.63 ± 1.03	52.56
Ca^{2+}	63.76 ± 1.44	113.11
Fe^{3+}	30.98 ± 0.88	54.96
Pb^{2+}	20.81 ± 0.46	36.92
Al^{3+}	27.47 ± 0.70	48.73
K^{+}	37.46 ± 0.46	66.45
